# Pelagic productivity and abundance of competitors modulate trophic niche segregation between large predators

**DOI:** 10.1098/rspb.2025.1809

**Published:** 2025-10-29

**Authors:** Elena Fernández-Corredor, Joan Navarro, Alba Fuster-Alonso, Joan Giménez, Salvador García-Barcelona, Lucía Rueda, David Macías, Marta Coll, Francisco Ramírez

**Affiliations:** ^1^ Institut de Ciències del Mar, Barcelona, Spain; ^2^ Departamento de Estadística e Investigación Operativa, Universidad de Valencia, Valencia, Spain; ^3^ Instituto Español de Oceanografía Centro Oceanográfico de Málaga, Fuengirola, Spain; ^4^ Ecopath International Initiative (EII), Barcelona, Spain

**Keywords:** swordfish, blue shark, shortfin mako, pelagic fish, isotopic niche, competition, stable isotopes, top predators, diet, fisheries

## Abstract

In the open ocean, large pelagic predators often share similar food resources and feeding grounds, probably avoiding competition and coexisting through niche partitioning. Building on this hypothesis, we combined spatial distribution data with isotopic niche metrics and diet reconstructions based on C and N stable isotopes to describe intra- and interspecific competition between three sympatric predators: swordfish (*Xiphias gladius*), blue shark (*Prionace glauca*) and shortfin mako (*Isurus oxyrinchus*). We then evaluated the role of biological (competitor abundance), environmental (pelagic productivity) and anthropogenic (fishing pressure) drivers in shaping competition metrics within and between species. Shortfin makos had a high isotopic niche overlap with blue sharks (>80%), feeding on similar prey. In the Mediterranean, the isotopic niche of swordfish was narrower than, and highly overlapped with, that of blue sharks, although diet estimates suggest that swordfish rely more on fish while blue sharks rely more on squid. On average, the potential for intraspecific competition was highest for swordfish and lowest for shortfin makos. Our results suggest that pelagic productivity and competitor abundance are key drivers of intra- and interspecific trophic niche segregation between large pelagic predators, respectively. They support the hypothesis that niche partitioning is reduced under a scenario of high resource availability.

## 1. Introduction

Coexisting, ecologically similar individuals often compete for limited resources [1]. However, competition (and ultimately competitive exclusion) can be mitigated through niche partitioning, a process by which individuals differentiate their use of resources [2]. This differentiation, involving fine-scale resource partitioning and spatial and/or temporal segregation, can occur within and between species [3] and serves as a key mechanism facilitating the coexistence of conspecifics and ecologically similar sympatric species [4,5]. Among those limited resources that might be partitioned, food is particularly critical due to its significant effects on growth, development and reproductive performance (e.g. [6]).

In both intra- and interspecific contexts, trophic niche width and overlap are determinants of competition dynamics [7,8]. The width of the trophic niche reflects the range of food resources exploited by individuals within species/populations and can vary with environmental heterogeneity and the presence of competitors [9,10]. When resources become limited in the environment, the degree of competition and the amount of overlap can be directly related [11]. High niche overlap between species can exacerbate competition and drive species towards specialization or broader generalist strategies over evolutionary time scales, depending on their relative abundance [12].

In the open ocean, large pelagic predators often share similar food resources and feeding grounds and are examples of ecologically similar species likely to coexist in highly exploited marine regions and potentially exhibit niche partitioning [13]. Many large pelagic species like pelagic sharks, tunas and billfishes are heavily exploited by longline fleets, with the northeast Atlantic and the Mediterranean Sea being two of their primary fishing grounds, and the Spanish fleet leading the total catches across these regions [14]. The main target species of these fleets are swordfish (*Xiphias gladius*) and albacore tuna (*Thunnus alalunga*). However, ecologically similar species such as the blue shark (*Prionace glauca*) and the shortfin mako (*Isurus oxyrinchus*) are also captured, with blue shark catches even exceeding those of swordfish [14]. While fisheries pose a significant threat to large pelagic species, they can also act as an additional competitor by removing prey and predators from the ecosystem, thereby altering food availability and trophic dynamics [15]. Furthermore, blue and shortfin mako sharks may compete with swordfish for the same resources, as pelagic fishes and squid constitute the main prey for all three species [16,17]. The abundance and availability of these prey species, which are also targeted by fisheries, are closely linked to oceanographic conditions such as marine productivity [18,19] and play a key role in shaping and partitioning trophic niches [9,20].

Understanding the complexity of these competitive interactions remains a significant challenge since an appropriate proxy is required to study both intra- and interspecific competition. In this context, stable isotope analysis (SIA) has emerged as a pivotal tool for quantitatively studying niche dynamics and resource partitioning among sympatric species (e.g. [13,21–23]). By integrating information on resource use and habitat through isotopic signatures (typically *δ*^13^C and *δ*^15^N), this approach provides both quantitative and qualitative insights into the trophic niche, visualized within a multivariate space: the isotopic niche [24,25]. The isotopic niche width can be used as a proxy for diet diversity, which probably shapes intraspecific competition. Simultaneously, isotopic niche overlap between coexisting species can be used to estimate the similarity of exploited resources, potentially reflecting interspecific competition when shared resources are limited [26,27]. On the other hand, environmental variables such as those related to oceanographic conditions can influence the isotopic niche width and overlapped area between species [13,28]. In addition to assessing isotopic niche overlap between competitors, stable isotope mixing models can be used to evaluate the relative similarity in exploiting specific prey resources. These models provide a framework for estimating prey consumption based on the isotopic ratios of predators and their potential prey [29]. This approach provides valuable proxies for evaluating the mechanisms underlying trophic competition and for predicting how shifts in ecosystem dynamics and fishing pressure may alter predator interactions and food web structure.

Most studies employing SIA have examined intra- and interspecific competition in isolation, with relatively few investigating both components simultaneously within ecological communities and across environmental gradients (e.g. [27]). Here, we describe the isotopic niche and diet of swordfish, blue shark and shortfin mako shark from the northeast Atlantic and the western Mediterranean Sea to unravel potential intra- and interspecific competition between these species in a heterogeneous seascape. By examining the isotopic niches of these species along a gradient of fishing pressure and environmental conditions, we aim to identify the drivers of competition and unravel the complex relationships between these top predators, their prey and the environment. We hypothesize that niche partitioning occurs between species and among conspecifics, driven by the *per capita* availability of primary prey through competition. Specifically, we expect that: (i) in areas of higher pelagic productivity, and therefore higher prey abundance, trophic niche partitioning may be reduced due to decreased competition; and (ii) an increase in the abundance of potential competitors—including conspecifics, competing species and fisheries—could diminish *per capita* food availability, intensifying competition and enhancing trophic niche partitioning.

## 2. Methods

### (a) Study species, area and data collection

We focused on three species of conservation concern: swordfish, blue shark and shortfin mako shark. Swordfish and blue sharks are classified as near threatened by the International Union for Conservation of Nature (IUCN) at the global level, whereas shortfin makos are considered endangered [30–32]. In the Mediterranean Sea, both shark species are classified as critically endangered [33,34].

**Figure 1.**
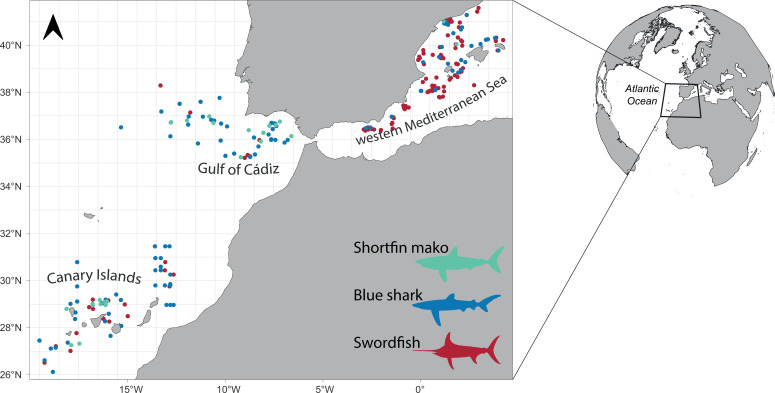
Study area and sampling locations along the western Mediterranean Sea and the northeastern Atlantic Ocean. Grey lines delineate a spatial grid of 1°×1°.

Swordfish (*n* = 253), blue shark (*n* = 187) and shortfin mako shark (*n* = 31) individuals were sampled between 2017 and 2019 by longline fishing vessels operating along the western Mediterranean Sea and the northeastern Atlantic Ocean (figure 1). Both basins, connected by the Strait of Gibraltar, exhibit distinct oceanographic, ecological and human impact characteristics [35–37], thus offering the opportunity to study the niche segregation of those species along a gradient of environmental conditions and human activities.

Shark fork length (FL, in cm) and swordfish lower jaw fork length (LJFL, in cm) were measured on board. White muscle samples of each individual were collected and frozen at −20°C until further analyses. Information related to technical characteristics and fishery strategies of longlines can be consulted in García-Barcelona *et al.* [38].

### (b) Environmental drivers, fisheries and abundance

Here, we evaluated the influence of surface chlorophyll-a concentrations (Chl) and the ‘Ocean Productivity available to Fish’ (OPFish) as proxies for prey availability [39], whereas the mixed layer depth (MLD) was used as a proxy for upper ocean dynamics that influence mesopelagic processesand oxygen minimum zones [13,40]. Chl and MLD monthly values for the 2015−2019 period were obtained from the EU Copernicus Marine Environment Monitoring Service. The OPFish integrates the horizontal gradient of Chl, the range of Chl content and the relative day length duration to refer to marine ecosystem feeding hotspots and use as a proxy of the potential fish production of pelagic species [41] and was obtained from Druon [42].

Spatially explicit estimates of cumulative fishing effort were obtained from Global Fishing Watch (GFW) [43], accessed in 2024 from the Google Earth Engine. We summed daily fishing records from commercial fisheries (trawlers, purse seiners, gillnets and longliners) to obtain spatially explicit cumulative values covering the 2015−2019 period.

*Per capita* food availability is influenced by the abundance of potential competitors, including both conspecifics and individuals of ecologically similar species. As a proxy of the relative abundance of the three target species, data from 2534 longline sets from the Spanish longline fleet targeting swordfish and albacore tuna along the study area between 2015 and 2019 were included in the analysis (IEO Observer Program). The catch per unit effort (nCPUE; number of individuals per thousand hooks) was standardized using generalized additive models (GAMs) with a negative binomial error distribution to account for overdispersion in the catch data (electronic supplementary material, Methods). The number of hooks was included as an offset variable. Latitude and longitude were incorporated using a tensor product smooth function to capture spatial variability in catch rates. The longline type was included as a fixed factor, while year, month and boat were treated as random effects to account for variability in sampling effort and fishing efficiency. Separate models were fitted for each species and basin (Mediterranean and Atlantic), resulting in five models (electronic supplementary material, table S1), with no model adjusted for shortfin mako in the Mediterranean Sea due to an excess of zero catch observations. These models were then used to predict the number of individuals caught per 1000 hooks for each grid cell and longline type, providing spatially explicit estimates of species relative abundance across the study area (table 1).

**Table 1.** List of explanatory variables used and their respective source and unit of measurement.

variables		source
Chl	chlorophyll concentration (mg m^−3^)	Copernicus
MLD	ocean mixed layer thickness (m)	Copernicus
OPFish	ocean productivity available to fish (% of daily favourable occurrence)	Druon [42]
fishing effort	cumulative fishing effort (h km^−2^)	Global Fishing Watch [43]
relative abundance	individuals of each species caught per 1000 hooks (nCPUE)	*this study*

White muscle tissue in large pelagic fishes reflects dietary intake over several months. To focus on broad-scale patterns of potential competition and reduce the influence of short-term variability, we standardized environmental variables (MLD, Chl and OPFish), fishing effort and relative abundances over a common temporal window (2015−2019). All layers were cropped to encompass the study area and adjusted to a common resolution of 1°×1° grid cells (electronic supplementary material, figure S1) using the *raster* R package v. 3.6-14 [44]. This spatial resolution was chosen to align with the movements of these vagile species while capturing the relevant spatial variability in competition and its relationships with the explanatory variables [39,45–47].

### (c) Stable isotope analyses

Prior to stable isotopic analysis, lipids were extracted with a chloroform/methanol (2:1) solution to correct for the differences in lipid content in the tissue on *δ*^13^C values [48]. For shark samples, the urea content was extracted by placing the samples immerse in deionized water in a sonication machine to remove the supernatant of urea, following Kim & Koch [49]. Samples were then dried at 60°C and ground to a fine powder. Analyses of *δ*^13^C and *δ*^15^N values were performed at the Laboratory of Stable Isotopes (LIE-EBD, Spain) as described in Fernández-Corredor *et al.* [17].

Interspecific differences in *δ*^13^C and *δ*^15^N values were tested using two-way permutational analysis of variance (PERMANOVA) tests on the Euclidean distance matrix, including the species and the area (western Mediterranean, South of Portugal/Gulf of Cádiz [hereafter Portugal/Cádiz], Canary Islands) as factors.

### (d) Isotopic niche partitioning

The isotopic niche was estimated using kernel utilization density estimators (KUDs; [50]) using the *rKIN* R package v. 1.0.2 [51]. Niche width and niche overlap between species were reported using the two-dimensional KUD of niche size at the 95% confidence level, which provides a good representation of irregularly distributed data [50]. The potential for interspecific competition was estimated through the isotopic niche similarity between the species by calculating the distance to the competitor’s centroid (CCD) within the isotopic niche space [27,52]. The CCD measures the dietary niche similarity of a species with its potential competitors in the *δ*^13^C *–δ*^15^N space and is defined as


$${\text{CCD}}_{i}=\frac{1}{N}\sum_{k=1}^{N}d\left(C_{i},\;C_{k}\right)\;,$$

where $d\left(C_{i},C_{k}\right)$ is the Euclidean distance between $C_{i}$ (the centroid of the species *i*) and $C_{k}$ (the centroid of competitor *k*), *N* is the total number of potential competitors and ${\text{CCD}}_{i}$ is the mean of Euclidean distances between the centroid of the species *i* and those of their competitors.

The intraspecific competition was estimated using the intraspecific trophic pressure index (ITP; modified from Pelage *et al.* [26]), calculated as


$${\text{ITP}}_{ij}=\log\left({\text{nCPUE}}_{ij}+1\right)/{\text{KUD}}_{ij},$$

where ${\text{nCPUE}}_{ij\;}$ is the standardized catch per unit effort (individuals of species *j*/1000 hooks) for the grid cell *i* and ${\text{KUD}}_{ij\;}$ is the KUD of niche size at the 95% confidence level for the species *j* for the grid cell *i*. Abundant species with a narrow dietary niche show high ITP values, suggesting they exert strong trophic pressure on a limited set of resources; while less abundant species with a broad dietary niche show lower ITP values, reflecting their use of diverse resources [53].

The CCD and the ITP were calculated based on the KUD at the grid cell level. We assessed the normality of the distribution of CCD and ITP values using the Shapiro–Wilk test. When normally distributed, we conducted a one-way ANOVA to evaluate differences between groups, and post hoc pairwise comparisons were performed using the Tukey honestly significant difference test. For metrics that did not meet the normality assumption, we employed the Kruskal–Wallis test or PERMANOVA. Pairwise comparisons were conducted using the pairwise Wilcoxon rank-sum test.

### (e) Competition metrics and relationship with drivers

To evaluate the effect of the environmental drivers and fishing pressure on inter- and intraspecific competition for each species, isotopic data were pooled over a 1°×1° grid and niche metrics were calculated for each cell. Models for ITP and CCD were fitted using GAMs with a gamma distribution including linear and additive relations with the drivers (table 1; electronic supplementary material, Methods). Latitude and longitude were incorporated using a tensor product smooth function to capture spatial variability in isotopic values. All predictor variables were checked for potential Spearman correlation, and Chl was excluded (corr > 0.8; electronic supplementary material, figure S2). The Akaike information criterion (AIC) and the deviance explained (DE) were used for model selection. For each competition metric, the model with higher DE and lower AIC was chosen to evaluate the effects of explanatory variables. GAMs were built using the *mgcv* R package v.1.9-1 [54].

### (f) Food resource partitioning

We ran species-specific Bayesian stable isotope mixing models (BSIMMs) based on *δ*^13^C and *δ*^15^N values to estimate the relative contribution of the potential prey items in the diet of the three species. BSIMMs were computed using the *MixSIAR* R package v. 3.1.12 [29] and run with a generalist type prior, three Markov chain Monte Carlo chains of 300 000 draws and a burn-in of 200 000 draws. The convergence of models was checked using Gelman-Rubin and Geweke diagnostics [29,55]. The diet-tissue discrimination factors (DTDF) were estimated following Caut *et al.* [56] (electronic supplementary material, table S2). The appropriate parametrization of the BSIMMs was validated through the simulation of mixing polygons, where all consumer data corrected for DTDFs should fall within the bounds of the mixing space [57,58]. Prey groups were selected according to the available literature to represent the main potential prey in the diet of the three species in the Atlantic and Mediterranean, and included fishes, squids, gelatinous organisms and large pelagic species [16,17,59]. Isotopic values of prey species were obtained from the literature and pooled into six groups [60] using a meta-analysis approach based on similar isotopic values and taxonomy (electronic supplementary material, table S3, figure S3, Methods). To minimize potential confounding effects with stable isotope baselines, we used region-specific prey values [47].

All statistical analyses were conducted using R v. 4.3.3 [61].

## 3. Results

### (a) Spatial distribution of the abundance

**Figure 2.**
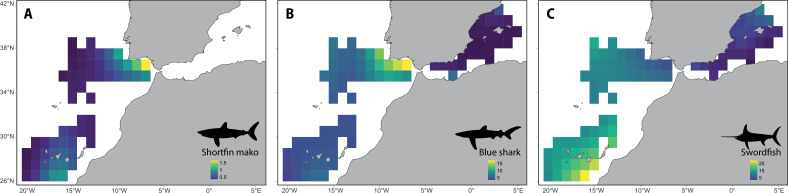
Spatial prediction of the abundance (nCPUE, individuals per 1000 hooks) for (A) shortfin mako, (B) blue shark and (C) swordfish between 2015 and 2019. Data are predicted within a 1°×1° grid.

The western Mediterranean showed the lowest relative abundance for the three species (figure 2). Shortfin mako was the least frequent species, with no model adjusted for the Mediterranean Sea due to a high number of zero catch observations. Shortfin makos had a higher nCPUE in the Gulf of Cádiz and the waters south of the Canary Islands. Blue sharks were more abundant in the Gulf of Cádiz and the southern waters of Portugal. Swordfish nCPUE was higher in the Atlantic, particularly in the waters south of the Canary Islands, than in the Mediterranean, where more individuals were caught in the northern part.

### (b) Isotopic niche and competition metrics

**Figure 3.**
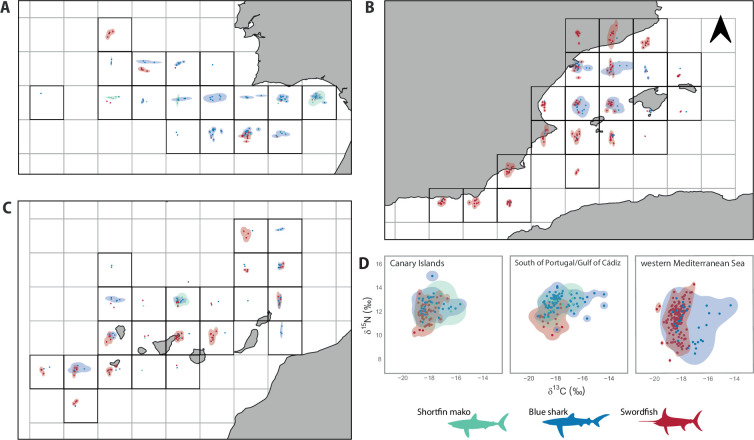
Isotopic niche for shortfin mako, blue shark and swordfish based on 95% kernel utilization densities. Isotopic niche is represented for each 1°×1° grid cell in (A) the South of Portugal/Gulf of Cádiz, (B) the western Mediterranean Sea and (C) the Canary Islands, and (D) summarized by area. The axes for each grid cell in (A–C) are the same as those in panel (D).

The *δ*^13^C and *δ*^15^N values differed between species, showing differences between regions (table 2). Overall, swordfish showed lower *δ*^13^C and *δ*^15^N values than blue and mako sharks (PERMANOVA *p* < 0.01; electronic supplementary material, table S4). Regarding the isotopic niche width, blue sharks showed the largest KUD in the Canary Islands (KUD area = 10.18‰²), followed by swordfish (7.5‰²) and shortfin makos (7.13‰²; figure 3D). In Portugal/Cádiz, blue sharks showed the largest KUD area (10.83‰²), followed by shortfin mako (8.8‰²) and swordfish (7.42 ‰²). The largest isotopic niche for both swordfish (KUD area = 10.47‰²) and blue shark (23.06‰²) was found in the western Mediterranean. Shortfin mako’s KUD area in the western Mediterranean was not calculated due to low sample size (*n* = 2).

**Table 2.** Mean values and standard deviation (s.d.) of length (FL: fork length, for sharks; LJFL: lower jaw fork length, for swordfish), *δ*^13^C and *δ*^15^N values for shortfin mako, blue shark and swordfish. *n*: number of samples.

species	area	*n*	FL/LJFL (cm)		*δ***^13^C**‰		*δ***^15^N**‰	
			mean	range	mean	s.d.	mean	s.d.
shortfin mako	Canary Islands	10	108.4	67−156	−17.87	0.66	11.91	0.70
	Portugal/Cádiz	19	130.2	72−189	−17.78	0.71	12.56	0.70
	W. Mediterranean	2	123.8	88−160	−16.69	0.55	13.08	1.05
blue shark	Canary Islands	50	159.0	93−237	−17.67	0.97	12.65	0.64
	Portugal/Cádiz	92	148.4	82−234	−17.72	0.99	12.87	0.64
	W. Mediterranean	45	118.8	43−218	−17.49	0.96	11.29	1.32
swordfish	Canary Islands	66	104.3	78−159	−18.25	0.48	11.94	0.77
	Portugal/Cádiz	27	103.4	80−114	−18.52	0.55	11.72	0.68
	W. Mediterranean	160	100.1	72−173	−18.26	0.46	11.41	1.24

Regional differences were observed in the isotopic overlap between the three species (see figure 3D; electronic supplementary material, table S5). The pairwise overlaps between species showed a higher isotopic overlap between mako and blue sharks than with swordfish. The percentage of overlap for swordfish isotopic niche was similar to both shortfin mako and blue shark, with a high overlap with blue sharks in the Mediterranean Sea (92%), while this overlap only represented 42% of the blue shark isotopic niche.

**Figure 4.**
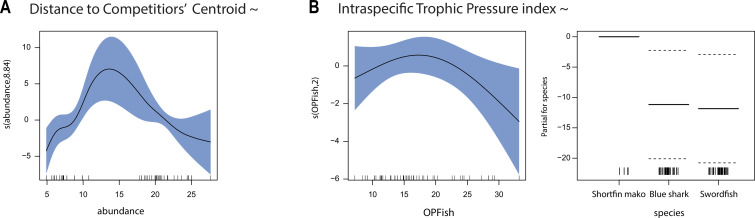
GAM partial effects of the explanatory variables for (A) the distance to the competitor’s centroid within the isotopic niche space (CCD) and (B) the intraspecific trophic pressure (ITP) index: panel on the left shows the partial effect of OPFish (smooth term) on ITP across all species, while panel on the right shows the partial effect of species (fixed term) on ITP. Shadowed areas represent 95% confidence intervals around the main effects.

Distances to the competitor centroid were shorter in the western Mediterranean (CCD = 1.13 ± 0.52 for blue sharks, and 1.17 ± 0.58 for swordfish) than in Portugal/Cádiz and the Canary Islands (ANOVA, *p* < 0.05). CCD for shortfin mako was higher in the Mediterranean (1.78 ± 0.83). The best-fitted model for CCD included the abundance of the three species and the spatial component, explaining 84.3% of the deviance (electronic supplementary material, table S6). Diagnostic plots suggested normality and homoscedasticity of the residuals (electronic supplementary material, figure S4). The distance to the potential CCD within the isotopic niche space increased with the abundance of the three species and decreased when intermediate abundances were reached (figure 4A).

On average, the potential level of intraspecific competition was higher in the swordfish (ITP = 0.75 ± 0.76) than in the blue shark (0.52 ± 0.65), with both species showing higher values than shortfin makos (0.08 ± 0.05; Kruskal–Wallis *p* < 0.01, pairwise Wilcoxon test *p* < 0.05). The best-fitted model for potential intraspecific competition included pelagic productivity, the species and the spatial component, explaining 52.7% of the deviance (electronic supplementary material, table S6). Diagnostic plots suggested normality and homoscedasticity of the residuals (electronic supplementary material, figure S4). Potential intraspecific competition intensity decreased as intermediate values of pelagic productivity were reached (figure 4B).

### (c) Food resource partitioning

**Figure 5.**
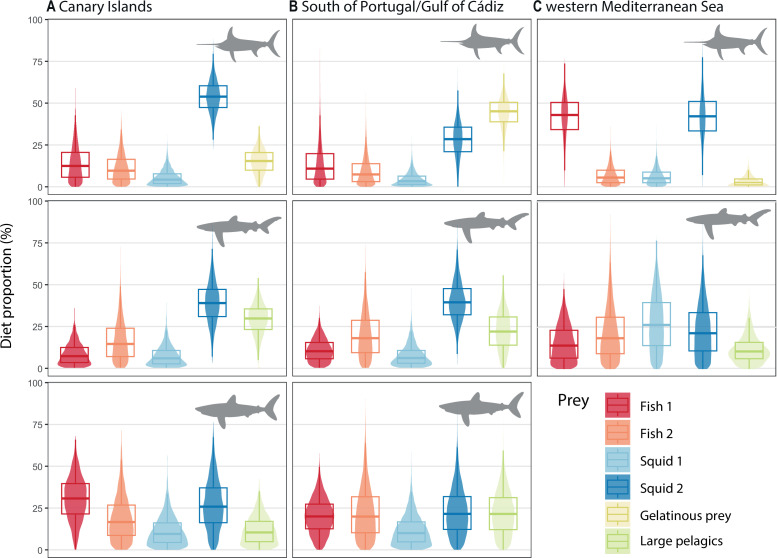
Relative contribution of prey to the diet of swordfish (top row), blue sharks (medium row) and shortfin makos (bottom row) from (A) the Canary Islands, (B) the South of Portugal/Gulf of Cádiz and (C) the western Mediterranean Sea obtained through Bayesian stable isotope mixing models (BSIMMs). The composition of each prey group is available in electronic supplementary material, table S3.

In the Canary Islands, squid 2 (cock-eyed squids) were the most consumed prey for the swordfish and blue shark (figure 5). Together with fish 1 (mesopelagic fishes), they contributed to almost 50% of the diet of the shortfin mako. Dietary estimates suggested a low contribution of gelatinous prey to swordfish diet except in Portugal/Cádiz, representing almost 50% of the contribution to the diet, and followed by squid 2 (cock-eyed squids). Blue sharks showed a similar diet for both Portugal/Cádiz and the Canary Islands, with squid 2 being the main prey (around 40% of their diet), followed by large pelagics. All prey groups showed similar contributions to the diet of shortfin mako in the Gulf of Cádiz, pointing at a generalist diet. Both sharks had higher fish contribution to their diet than swordfish in the Gulf of Cádiz and the Canary Islands. In the western Mediterranean, the swordfish diet was mainly based on fish 1 (mesopelagic fishes) and squid 2 (midwater and angel squid), while the estimated diet for blue sharks had a higher contribution of squid 1 (ommastrephids) and fish 2 (gadiforms and pelagic fish). Blue sharks showed the lowest assimilation of fish 1 (mesopelagic fishes) in both the Mediterranean and Atlantic Ocean. Gelman-Rubin and Geweke diagnostics suggested a satisfactory fit for the eight models (electronic supplementary material, table S7).

## 4. Discussion

Understanding the mechanisms that shape the trophic niche and facilitate the coexistence of ecologically similar species is a complex challenge, particularly in the open ocean, where direct observations and samples are difficult to obtain. Adopting an integrated approach, considering external drivers such as environmental variables and fishing pressures, can offer valuable insights into the processes that regulate competition between conspecifics and ecologically similar species. In this study, we combined spatial distribution data, isotopic niche metrics and dietary reconstructions to describe spatial patterns of intra- and interspecific potential competition. This approach allowed us to understand how external drivers influence niche partitioning both between and within three ecologically similar sympatric large predatory fishes. Our results suggest that the pelagic productivity and the abundance of potential competitors are key drivers of intra- and interspecific trophic niche segregation between large sympatric pelagic predators.

### (a) Isotopic niche and food resource partitioning

The isotopic values and ranges observed between the three species suggest similar trophic ecologies [62] with some degree of niche partitioning. Both shortfin mako and blue shark showed higher *δ*^13^C values than swordfish, suggesting higher use of nearshore productive regions. In the Atlantic, swordfish showed lower *δ*^15^N values than shortfin mako and blue sharks, which could be explained by swordfish feeding on smaller prey with lower trophic levels than sharks [63]. This pattern is also highlighted in our diet estimates, with swordfish having higher consumptions of gelatinous organisms and mesopelagic fish. Shortfin mako sharks previously showed higher *δ*^15^N values and higher trophic positions than blue sharks in the Pacific [64,65]. However, in the Atlantic, blue sharks showed the highest *δ*^15^N values of the three species, which could be explained by their scavenger behaviour, occasionally feeding on marine mammal carcasses [63,66]. Higher *δ*^15^N values for swordfish compared with blue sharks in the western Mediterranean can result from a higher exploitation of deeper waters [67], as inferred by a higher consumption of mesopelagic fishes in this area.

The three studied species are known to feed on multiple prey types and exploit diverse trophic resources and habitats, consequently occupying a broad niche space [62,68]. The opportunistic behaviour of blue sharks is also reflected by their consistently wider isotopic niche along the studied area, indicative of a more generalist diet than swordfish and shortfin mako sharks. However, previous studies in the Pacific found a wider isotopic niche for shortfin mako, consuming a wider variety of prey than blue sharks, which were feeding mainly on the most abundant crustacean and squid species in the studied area, pointing to a more specialized diet [65]. A wider isotopic niche can result from trophic plasticity and adaptation to environmental heterogeneity, as well as from limited *per capita* prey availability [69]. In the Mediterranean, swordfish and blue sharks’ isotopic niches were wider, suggesting more dietary variability in prey size/type in this area. Although the abundance of the three potential competitors is lower than in the Atlantic, *per capita* prey availability is probably limited. The western Mediterranean is a highly exploited area [70,71] where fisheries are removing prey and can act as competitors themselves [15]. This scarcity may lead to heightened interspecific competition, expanding trophic and isotopic niche breadths [72]. In more productive areas such as the eastern tropical Pacific, no overlap was found between swordfish and blue shark or shortfin mako [62,68]. A lack of overlap was also reported for the three species in the northeast central Pacific [68]; however, blue shark and shortfin mako showed highly overlapping isotopic niches in the Ecuadorian Pacific area [62]. Here, we found that shortfin mako sharks had a high isotopic niche overlap with blue sharks (over 80%), with similar prey consumption obtained from the mixing models, suggesting the potential for competition for resources.

Although the studied species feed mainly on squid and pelagic fishes [16,17,73], regional variations in prey contribution proportions can lead to the observed isotopic differences. Regarding fish prey, swordfish showed higher consumption of mesopelagic fishes in the Mediterranean, while blue sharks seem to prefer pelagic fishes. We found a high overlap between swordfish isotopic niche and blue sharks in the western Mediterranean (more than 90%), suggesting that swordfish may share more food resources with blue sharks than in the Atlantic. However, the diet estimates suggested that these two species rely on different prey groups, with swordfish showing higher consumption of mesopelagic fish and midwater and angel squids, while blue sharks showed higher proportions of pelagic fishes and ommastrephids. Previous studies showed indications of trophic niche partitioning between the two shark species on the northeastern Atlantic, with fish being the main prey for shortfin makos, and blue sharks’ diet being dominated by cephalopods [16]. This example of food partitioning among coexisting species often reduces niche overlap and helps reduce interspecific competition [74].

### (b) Competition metrics and drivers

When the abundance of competitors is low, competition for resources is expected to be at its lowest, enabling an extensive niche overlap [12]. Under these conditions, generalist species such as those in this study are expected to feed on whatever is abundant, resulting in similar isotopic values and low isotopic distance between them. Accordingly, our results suggest that isotopic differences between swordfish and sharks were minimal (i.e. low CCD) in areas of low potential competitor abundances (figure 4A and Box 1A). As the abundance of competitors increased, the isotopic distance between species increased (i.e. high CCD), suggesting a possible change in trophic behaviour through the diversification of prey items. This behaviour can be interpreted as an example of food partitioning where species use different resources to avoid competition, which results in a higher isotopic distance [9]. However, as potential competitor abundance increased, the isotopic distance decreased, suggesting that in the areas with the highest abundance of competitors, the resource partitioning decreased. This could be explained by competitors aggregating in areas where food resources are abundant, leading to isotopic similarities due to similar prey consumption.

Box 1. **Conceptual framework based on the modelled relationships between competitor abundance and isotopic distance to competitor centroid (CCD), and pelagic productivity and intraspecific competition pressure (ITP).**

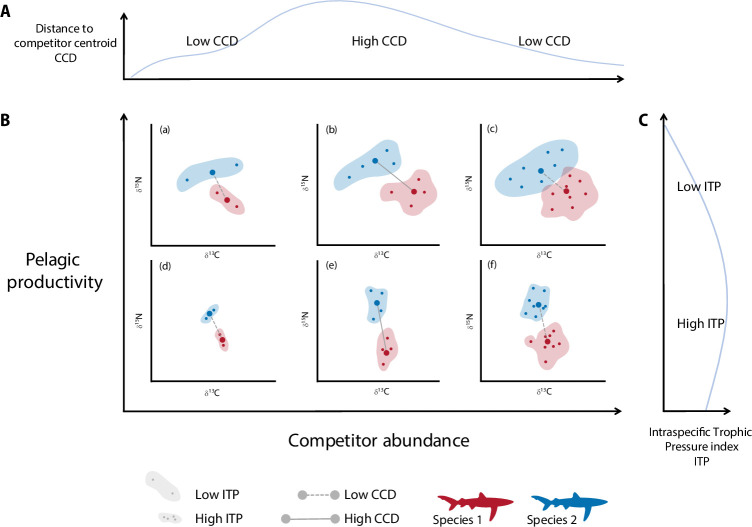

This figure illustrates the relationship between the different drivers and the different metrics used in this study to evaluate isotopic niche metrics as a proxy of intra- and interspecific competition. The blue lines represent the output from the GAMs showing (A) how the distance between centroids (*y*-axis) changes with competitor abundance (*x-*axis) and (C) how the intraspecific trophic pressure (*x*-axis) changes with pelagic productivity (*y*-axis). Panel B presents a matrix summarizing the interaction between pelagic productivity and competitor abundance, highlighting their effects on ITP and CCD. Each point in (B) represents individual isotopic values for species 1 (red) and species 2 (blue), bigger points denote the centroid for each species’s isotopic niche, represented by the shadowed area, and the grey lines represent the distance between the centroids. The matrix is divided into six quadrants, representing various combinations of competitor abundance and pelagic productivity:

(a) High pelagic productivity, low competitor abundance: both ITP and CCD were low, reflecting a scenario where abundant resources minimize competition and niche segregation is not necessary.
(b) High pelagic productivity, intermediate competitor abundance: low ITP and high CCD characterize this scenario. Resource availability allows species to diversify their niches, increasing isotopic distance among species and maintaining the intraspecific pressure low.
(c) High pelagic productivity, high competitor abundance: under conditions of high resource availability and competitor abundance, intraspecific competition may become more prevalent than interspecific competition. In such cases, species may maintain broader isotopic niches (low ITP) to reduce overlap with conspecifics, even if it results in greater isotopic overlap with other species (low CCD).
(d) Low pelagic productivity, low competitor abundance: with fewer competitors and low productivity, competition persists due to resource scarcity, with limited isotopic niche separation (low CCD) and high competition among conspecifics (high ITP).
(e) Low pelagic productivity, intermediate competitor abundance: the moderate abundance of competitors may encourage niche diversification, increasing isotopic distance between species (high CCD) while maintaining intraspecific competition pressure. This could be driven by density-dependent processes, where the most direct competitor is another individual with identical ecological requirements (high ITP).
(f) Low pelagic productivity, high competitor abundance: limited resources probably intensify competition, reducing isotopic differentiation among conspecifics and competitors due to feeding on the same prey. This scenario results in high ITP and low CCD.

In the open ocean, the distribution and the availability of pelagic resources over time and space are key factors shaping top predators’ feeding behaviour [63]. Our results point to pelagic productivity as the main driver shaping the intraspecific competition and support the hypothesis of niche partitioning between conspecifics being reduced (i.e. low ITP) under a high resource availability scenario (Box 1C). Additionally, we anticipated that an increase in the abundance of competitors would enhance trophic niche segregation by reducing *per capita* food availability. However, we did not find a relationship between intraspecific trophic pressure and fishing effort, and found the highest isotopic distances in areas of intermediate predator abundance, although the limited number of intermediate values increased uncertainty in the observed peak (figure 4A).

The different scenarios illustrating how isotopic niche dynamics are influenced by pelagic productivity and competitor abundance according to our results (see Box 1) suggest that at both productivity scenarios, our indicators exhibit similar variations for both competitor abundance extremes (i.e. low ITP and low CCD were found for the high pelagic productivity scenario at the lowest and highest competitor abundances). This limitation indicates that using only these two metrics may not be sufficient to comprehensively understand niche dynamics. It is likely that additional factors and mechanisms, such as behavioural plasticity, prey switching or fine-scale habitat use, are influencing niche partitioning in ways not captured by isotopic metrics alone. Thus, our inferences should be viewed as conservative, and future studies should consider integrating complementary approaches, such as tagging studies (e.g. [46]), to gain a more holistic perspective on competitive interactions and resource use.

### (c) Limitations and broader implications

We examined trophic niche and food resource partitioning between ecologically similar species and conspecifics using isotopic niches and diet reconstructions through mixing models. White muscle isotopes reflect diet over several months, offering an integrated summary of trophic interactions but limiting fine-scale resolution. Moreover, while using temporally averaged pelagic productivity values helped capture broad-scale influence on competition, it does not account for potential seasonal variability. Nevertheless, the inclusion of pelagic productivity and potential competitor abundance significantly improved model performance over the null models, indicating their relevance in shaping ITP and CCD values, respectively. Our findings indicate that *per capita* food availability, probably driven by pelagic productivity and potential competitor abundance, plays a relevant role in shaping niche partitioning. However, we acknowledge that niche partitioning extends beyond the trophic dimension, encompassing other ecological and behavioural factors, including fine-scale temporal and spatial segregations (e.g. seasonal and diel variability in diving behaviour [13]). Despite overlapping horizontal habitats, both shark species undertake vertical movements up to 300 m during the day, returning to the surface at night, whereas swordfish display more pronounced vertical movements, spending daylight hours below the mixing layer and surfacing at night, as inferred by their high consumption of mesopelagic prey [75–78]. Future research could leverage this framework to integrate these other dimensions, providing a more comprehensive understanding of niche partitioning between ecologically similar species and disentangling finer temporal dynamics.

Our modelling approaches predict that blue sharks were particularly abundant in the Gulf of Cádiz and the southern waters of Portugal, thus aligning with findings from previous tagging studies [79,80]. Model predictions also reveal higher abundances of shortfin mako sharks in the Gulf of Cádiz and the southern waters of the Canary Islands, as described in [78]. Our predictions for swordfish are consistent with previous research on this species and suggest higher abundances around the Canary Islands [81]. We also provide the first assessments of swordfish distribution for the western Mediterranean Sea, despite the high commercial value of this species and the declining trends of this stock in the Mediterranean over the last 50 years [82]. The reduced populations of the three species in the Mediterranean Sea, with shortfin makos nearly absent in the western part, make them more vulnerable to stochastic ecological stressors [4] and highlight their vulnerability in the Mediterranean region. In this context, we could not estimate the diet of shortfin mako sharks in the Mediterranean due to insufficient samples. For the Canary Islands, while 10 samples for shortfin mako were available, larger sample sizes would provide more robust diet inferences.

Understanding how resource use and niche partitioning have shifted in response to historical variability can provide insights into how predators will respond to future environmental changes [83]. According to our results, if global change, including fishing and climatic pressures, leads to declining prey abundance, interspecific competition will probably increase and may impact less opportunistic species more severely [84]. Further prey reductions may increase intraspecific pressure, driving shifts in feeding behaviour and expanding niches, potentially causing greater overlap and interspecific competition. Those may explain the low numbers of shortfin mako and blue sharks in the Mediterranean, where competitive pressures together with the slower intrinsic rates of population growth of sharks may favour swordfish [85]. On the other hand, changes in prey abundance, particularly of squid, the main prey for the three species, are expected to be spatially heterogeneous [86]. In areas of high trophic overlap of predators, prey changes will potentially impact all species, while in regions where the overlap is lower, segregation could allow ecologically redundant species, less affected by the changes, to compensate for the ecological roles of more impacted species, potentially buffering ecosystem-level effects.

In conclusion, our study highlights the interplay between resource use, competition and oceanic productivity among large pelagic fishes in the Mediterranean and the northeast Atlantic. These pressures may intensify intra- and interspecific competition, potentially favouring species with adaptive advantages such as opportunistic feeding or rapid reproductive rates. Understanding these interactions becomes crucial for predicting population trends and developing effective conservation strategies that address both direct and indirect consequences of alterations in food availability.

## Data Availability

The data that support the findings of this study and the code used are openly available in Zenodo [87] and GitHub (https://github.com/ElenaFCorr/Trophic-niche-segregation-among-large-pelagic-predators). Supplementary material is available online [88].
